# A novel method for eliciting foot care preferences in juvenile idiopathic arthritis: a discrete choice experiment

**DOI:** 10.1186/1757-1146-4-S1-P25

**Published:** 2011-05-20

**Authors:** Gordon J Hendry, Janet Gardner-Medwin, Debbie E Turner, Jim Woodburn, Paula K Lorgelly

**Affiliations:** 1 School of Biomedical and Health Sciences, University of Western Sydney, Sydney, Locked Bag 1797, Australia; 2 Glasgow Caledonian University, Glasgow, G4 0BA, UK; 3 University of Glasgow, Glasgow, G12 8QQ, UK; 4 Monash University, Victoria, 3800, Australia

## Background

The management of foot problems in JIA is complex and requires a multidisciplinary health professional input. However little is known about patients’ and parents’ preferences for attributes of foot care. Incorporating individuals’ preferences into future foot-care provision may improve satisfaction, treatment adherence, and thus clinical efficacy. The aim of this study was to compare the relative importance of foot care attributes to parents of children/adolescents with JIA using an established stated preference method, a discrete choice experiment (DCE).

## Methods

A DCE questionnaire consisting of 18 paired hypothetical clinical scenarios was developed using qualitative methods and designed according to published design efficiency criteria. The attributes explored were: levels of pain (pain); mobility; ability to perform activities of daily living (ADL); waiting time for initial consultation (wait); referral route (route); and ability to wear desired footwear (footwear). ‘Cost to self’ (cost) was included as an attribute to estimate parents’ valuations of each alternative attribute, known as their willingness-to-pay (WTP). The DCE was self-completed by parents (n=40) of children/adolescents with JIA and disease-related foot problems. Data were analysed using a conditional logit regression model.

## Results

Each attribute’s regression coefficients (β) were statistically significant (p<0.01) except cost (β=0.002, p=0.118), suggesting that all attributes, except cost, had an impact on parents’ preferences. The magnitudes of the coefficients indicate that the order of importance (that is strength of preference) for each attribute was: ADL (β=1.65), pain (β=1.20), mobility (β=1.12,), footwear (β=1.05), route (β=0.53) and wait (β=-0.05). The sign of the β values suggests that parents preferred: a reduction in pain, improvements in mobility, the ability to perform ADL, and the ability to wear desired footwear; referral to a multi-disciplinary foot-care programme; and reduced waiting time. The insignificance of the cost attribute means that WTP values could not be estimated.

## Conclusions

In terms of foot care service provision for children with JIA, parents appear to prefer improvements in health outcomes over non-health outcomes and service process attributes. An interesting finding of this study, relative to other DCE studies, is that cost does not appear to impact on parents’ preferences, suggesting that parents are comfortable paying to improve their child’s health.

